# The Temperature-Dependent Expression of the High-Pathogenicity Island Encoding Piscibactin in *Vibrionaceae* Results From the Combined Effect of the AraC-Like Transcriptional Activator PbtA and Regulatory Factors From the Recipient Genome

**DOI:** 10.3389/fmicb.2021.748147

**Published:** 2021-11-19

**Authors:** Marta A. Lages, Manuel L. Lemos, Miguel Balado

**Affiliations:** Department of Microbiology and Parasitology, Institute of Aquaculture, Universidade de Santiago de Compostela, Santiago de Compostela, Spain

**Keywords:** fish pathogens, virulence factors, piscibactin, high pathogenicity island, aquaculture, *Vibrio anguillarum*, horizontal gene transfer

## Abstract

The high-pathogenicity island *irp*-HPI is widespread among *Vibrionaceae* encoding the piscibactin siderophore system. The expression of piscibactin genes in the fish pathogen *Vibrio anguillarum* is favored by low temperatures. However, information about the regulatory mechanism behind *irp*-HPI gene expression is scarce. In this work, in-frame deletion mutants of *V. anguillarum* defective in the putative regulators AraC1 and AraC2, encoded by *irp*-HPI, and in the global regulators H-NS and ToxRS, were constructed and their effect on *irp*-HPI gene expression was analyzed at 15 and 25°C. The results proved that only AraC1 (renamed as PbtA) is required for the expression of piscibactin biosynthesis and transport genes. PbtA inactivation led to an inability to grow under iron restriction, a loss of the outer membrane piscibactin transporter FrpA, and a significant decrease in virulence for fish. Inactivation of the global repressor H-NS, which is involved in silencing of horizontally acquired genes, also resulted in a lower transcriptional activity of the *frpA* promoter. Deletion of *toxR-S*, however, did not have a relevant effect on the expression of the *irp*-HPI genes. Therefore, while *irp*-HPI would not be part of the ToxR regulon, H-NS must exert an indirect effect on piscibactin gene expression. Thus, the temperature-dependent expression of the piscibactin-encoding pathogenicity island described in *V. anguillarum* is the result of the combined effect of the AraC-like transcriptional activator PbtA, harbored in the island, and other not yet defined regulator(s) encoded by the genome. Furthermore, different expression patterns were detected within different *irp*-HPI evolutionary lineages, which supports a long-term evolution of the *irp*-HPI genomic island within *Vibrionaceae.* The mechanism that modulates piscibactin gene expression could also be involved in global regulation of virulence factors in response to temperature changes.

## Introduction

Bacteria of the genus *Vibrio* are among the most predominant infectious agents threatening marine wildlife and aquaculture (Toranzo et al., 2005). *Vibrio anguillarum* is the etiological agent of classical vibriosis in fish, a typical hemorrhagic septicemia that causes high mortalities and economic losses in aquaculture worldwide (Toranzo et al., 2017). Increments in sea water temperature are associated with the proliferation of *Vibrio* species (Maeda et al., 2003), and hence, the subsequent occurrence of fish disease outbreaks (Le Roux et al., 2015). However, *V. anguillarum* is also able to cause vibriosis at cold temperatures (5–18°C) (Austin and Austin, 2007; Bellos et al., 2015; Ma et al., 2017). Numerous factors including motility, chemotaxis, LPS, extracellular products with hemolytic and proteolytic activities, and several iron-uptake systems have a role in *V. anguillarum* virulence (Rodkhum et al., 2006; Li and Ma, 2017; Toranzo et al., 2017). *V. anguillarum* adjusts the expression of some of these virulence factors by unknown mechanisms, responding to environmental signals such as iron levels and temperatures (Denkin and Nelson, 1999; Crisafi et al., 2014; Lages et al., 2019).

Bacteria possess tools to silence the expression of horizontally acquired genes. H-NS, an important global repressor of transcription in Gram-negative bacteria, functions in the process of xenogenetic silencing and also in the regulation of temperature-dependent gene expression (Stoebel et al., 2008; Prajapat and Saini, 2012; Mou et al., 2013). Another conserved global regulator is ToxR, which functions as a sensor and signal transducer controlling the expression of multiple virulence genes (ToxR regulon) in response to environmental cues (Skorupski and Taylor, 1997). Conversely, horizontally acquired DNA usually contains genes encoding transcriptional regulators that promote their own expression (Stoebel et al., 2008).

**FIGURE 1:**
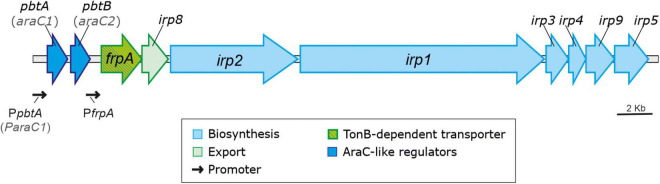
*V. anguillarum* piscibactin operon contained within the *irp*-HPI genomic island.

Production of siderophores is a key virulence factor for most bacterial pathogens including *V. anguillarum* (Miethke and Marahiel, 2007; Balado et al., 2018; Kramer et al., 2020). Highly virulent strains of *V. anguillarum* can simultaneously synthesize two siderophores: vanchrobactin and piscibactin (Balado et al., 2018). Vanchrobactin is considered the ancestral siderophore system of *V. anguillarum* since it is encoded by a chromosomal gene cluster (*vab* genes) that is widespread in all *V. anguillarum* isolates either environmental or pathogenic (Balado et al., 2006). The synthesis and transport of piscibactin is encoded by a high-pathogenicity island (*irp*-HPI element) (Osorio et al., 2015; Balado et al., 2018). In addition, *irp*-HPI encodes two conserved AraC-like regulators not studied so far (Figure 1), which are among the most upregulated genes of *V. anguillarum* when temperature decreases (Lages et al., 2019). This HPI was firstly identified within a plasmid in *Photobacterium damselae* subsp. *piscicida*, for which it is also a key virulence factor (Osorio et al., 2006, 2015). However, recent works showed that it is widespread among many species of the *Vibrionaceae* family, including relevant animal pathogens of the Splendidus and Harveyi clades and human pathogens like *V. cholerae* (Thode et al., 2018).

Siderophore production in *V. anguillarum* is balanced in a temperature-dependent manner since piscibactin genes have a dual requirement for iron starvation and low temperatures (<20°C) to be significantly expressed (Balado et al., 2018; Lages et al., 2019). Thus, the acquisition of *irp*-HPI seems to play a role in the adaptation of *V. anguillarum* to changing environments enhancing niche flexibility and enabling it to infect cold- and warm-water adapted fish (Balado et al., 2018; Lages et al., 2019).

In this work, we analyzed the effect of the two AraC-like regulators, *araC1* and *araC2*, contained in *irp*-HPI, and the global regulators H-NS and ToxR-S on the temperature-dependent expression of piscibactin genes in *V. anguillarum*. The results showed that AraC1 is the main transcriptional regulator that modulates the expression of the siderophore piscibactin system. In addition, although the global regulators, ToxR-S and H-NS, do not have major effects on *irp*-HPI expression, they indirectly intervene in the regulatory circuit of this genomic island through the modulation of the *frpA* promoter activity. Thus, the temperature-dependent expression pattern of *irp*-HPI genes results from the combined effect of regulatory factor(s) encoded outside the genomic island and the AraC1 transcriptional regulator within the genomic island.

## Materials and Methods

### Bacterial Strains, Plasmids, and Media

The bacterial strains and plasmids used in this work are listed in Table 1. *V. anguillarum* strains were grown at 25°C or 15°C in tryptic soy agar (TSA) or broth (TSB) (Cultimed) supplemented with 1% NaCl. *Escherichia coli* strains were grown in Luria-Bertani broth or agar (Cultimed) at 37°C. When required, antibiotics were added at the following final concentrations: kanamycin 50 μg ml^–1^, ampicillin sodium salt 60 μg ml^–1^ or 100 μg ml^–1^, and gentamycin 15 μg ml^–1^.

**TABLE 1:** Strains and plasmids used in this study.

**Strain or plasmid**	**Relevant characteristics**	**References**
***Vibrio anguillarum***		
MB14	RV22 with in-frame deletion of *vabF* gene	Balado et al., 2006
MB203	RV22 with in-frame deletion of *vabF* and *irp1* genes	Balado et al., 2018
ML168	RV22 with in-frame deletion of *vabF* and *pbtA* (*araC1*) genes	This study
ML136	RV22 with in-frame deletion of *vabF* and *pbtB* (*araC2*) genes	This study
ML293	RV22 with in-frame deletion of *vabF* and *h-ns* genes	This study
ML270	RV22 with in-frame deletion of *vabF* and *toxR-S* genes	This study
***Photobacterium damselae* subsp. *piscicida***		
DI21	Piscibactin producer strain	Toranzo et al., 1991
***E. coli***		
DH5α	Cloning strain	Laboratory stock
S17-1-*λpir*	RP4 (Km::Tn7, Tc::Mu-1) *pro-82 λpir recA1 end A1 thiE1 hsdR17 creC510*	Herrero et al., 1990
**Plasmids**		
pWKS30	Low-copy number cloning vector	Wang and Kushner, 1991
pNidKan	Suicide vector derived from pCVD442	Mouriño et al., 2004
pHRP309	Low-copy number *lacZ* reporter plasmid, *mob* Gm^*r*^	Parales and Harwood, 1993
pSEVA651	*mob* Gm^*r*^	Martínez-García et al., 2020
pMB276	*frpA* promoter (P*frpA*) fused to a promoterless *lacZ* gene in pHRP309	Balado et al., 2018
pMB277	*pbtA* promoter from *V. anguillarum* (P*pbtA*_*ang*_) fused to a promoterless *lacZ* gene in pHRP309	Balado et al., 2018
pLP9	*frpA* promoter from *P. damselae* subsp. *piscicida* (P*frpA*_*pdp*_) fused to a promoterless *lacZ* gene in pHRP309	This study
pLP28	*pbtA* promoter from *P. damselae* subsp. *piscicida* (P*pbtA*_*pdp*_) fused to a promoterless *lacZ* gene in pHRP309	This study
pML247	*pbtA* cloned in pSEVA651	This study

### Construction of *araC1*, *araC2*, *h-ns*, and *toxR-S* Defective Mutants by Allelic Exchange and Gene Complementation

In-frame deletions of *araC1*, *araC2*, *h-ns*, and *toxR-S* were constructed by allelic exchange in *V. anguillarum* RV22 strain in a Δ*vabF* background (impaired to synthesize vanchrobactin) as previously described (Balado et al., 2006). The flanking regions of each gene were amplified by PCR and cloned into the vector pWKS30; the resulting constructions were ligated into the suicide vector pNidKan. The resulting plasmid was conjugated with RV22Δ*vabF* strain and selected based on ampicillin and kanamycin resistance. A second event of recombination was performed, and the mutants were selected based on sucrose (15%) resistance. A PCR was performed to confirm the allelic exchange event. This process led to the formation of *V. anguillarum* mutant strains RV22Δ*vabF*Δ*araC1*, RV22Δ*vabF*Δ*araC2*, RV22Δ*vabF*Δ*h-ns*, and RV22Δ*vabF*Δ*toxR*-S. For *araC1* mutant complementation, *araC1* was amplified by PCR, cloned into the vector pSEVA651 in *E. coli* S17-1 λpir, and mobilized to the appropriate mutant strain by conjugation. To restore the original phenotype, the WT genes *h-ns* and *toxR*-S were cloned into the suicide vector pNidKan and the complementation was accomplished as indicated above for the construction of mutants. The oligonucleotides used are listed in Supplementary Table 1.

### Growth Ability and Siderophore Production Assay in Iron-Deficient Conditions

Growth ability assays were performed in CM9 medium supplemented with 10 μM FeCl_3_ to achieve iron excess or with 25 or 75 μM 2,2′-dipyridyl (TCI) to achieve iron deficiency. *V. anguillarum* strains RV22Δ*vabF*, RV22Δ*vabF*Δ*araC1*, RV22 Δ*vabF*Δ*araC2*, RV22Δ*vabF*Δ*h-ns*, RV22Δ*vabF*Δ*toxR*-S, and complemented strains were grown overnight in TSB-1. Each culture was adjusted to an OD_600_ = 0.5 and a 1:50 dilution was inoculated in CM9 medium. The resulting cultures were incubated at 15°C with shaking at 120 rpm. Growth was recorded after 48 h.

Bacterial cultures grown in CM9 medium with 25 μM 2,2′-dipyridyl and at an OD_600_ ∼ 0.8 were used to measure siderophore production with the chrome azurol-S (CAS) liquid assay (Schwyn and Neilands, 1987). Briefly, supernatants were obtained by pelleting bacterial cells using centrifugation and equal volumes of these supernatants were incubated with the CAS reagent at room temperature for 15 min. The quantification was performed by measuring A_630_ in a spectrophotometer (Hitachi).

### Transcriptional Fusions and β-Galactosidase Assays

The regions immediately upstream of *Photobacterium damselae* subsp. *piscicida* (DI21 strain) *araC1* and *frpA* genes were amplified by PCR and fused to a promoterless *lacZ* gene in the low copy number plasmid pHRP309. This process leads to the construction of plasmid pLP28 and plasmid pLP9 carrying constructs *araC1_*p*__*dp*_::lacZ* (P*araC1_*p*__*dp*_*) and *frpA_*pdp*_::lacZ* (P*frpA*_*pdp*_), respectively. Plasmids pMB277 and pMB276 carrying the lacZ fusions of *V. anguillarum araC1_*a*__*ng*_::lacZ* and *frpA_*ang*_::lacZ* (P*araC1_*a*__*ng*_* and P*frpA*_*ang*_ promoters) were previously constructed (Balado et al., 2018). The constructs were mobilized from *E. coli* S17-1 λpir to *V. anguillarum* (RV22Δ*vabF* and its derivative mutants) and *P. damselae* subsp. *piscicida* (DI21 strain) by conjugation. The presence of the promoters was confirmed by PCR. The resulting *V. anguillarum* strains and *P. damselae* subsp. *piscicida* carrying the promoter fusions were grown under weak iron restriction using CM9 medium supplemented with 25 μM 2,2′-dipyridyl. When the bacterial cultures reached an OD_600_ = 0.3, the β-galactosidase activities were measured by the method of Miller (1992). The results shown are means of three independent experiments.

### Western Blot Analysis

Overnight cultures of RV22Δ*vabF* and RV22Δ*vabF*Δ*araC1* were grown in TSB-1 and adjusted to OD_600_ = 0.5. A 1:50 dilution was inoculated in 10 ml CM9 medium supplemented with 25 μM 2,2′-dipyridyl. As the bacterial cultures reached an OD_600_ = 0.8, they were pelleted at 4,000 rpm, for 30 min at 4°C. The pellet was resuspended in 5 ml of 10 mM Tris-HCl and 0.3% NaCl, pH 8.0. Cellular disruption was accomplished by sonication on ice (five cycles of 30 s). Then, the samples were centrifuged at 4,000 rpm for 30 min at 4°C to eliminate the cellular debris. For the isolation of membrane proteins, 1% sarkosyl was added to the supernatant and incubated at room temperature for 30 min. The samples were centrifuged at 40,000 rpm for 30 min at 4°C and the pellet was resuspended in 20 μl of water. The samples were mixed 1:1 with SDS-PAGE loading buffer and loaded into a 12% polyacrylamide gel. After the separation by SDS-PAGE, the proteins were transferred onto a PVDF membrane as previously described. The membrane was blocked in blocking buffer (5% skim milk in TBST, Tris Buffered Saline with Tween 20) for 1 h at room temperature with shaking. The membrane was incubated overnight in the primary antibody solution against the target protein (1:10,000 dilution of the anti-FrpA antibody). After rinsing the membrane with TBST, it was incubated with the secondary antibody (1:10,000 anti-rabbit IgG HRP conjugate antibody) for 1 h at room temperature with shaking. The signal was detected using the Clarity^TM^ Western ECL substrate (Bio-Rad). Rabbit polyclonal antibodies against the external loop 6 (short peptide PGGFSPAPRSSGDKNGYSP) of FrpA (anti-FrpA) were purchased from GenScript.

### Fish Virulence Assays

Experimental infections were performed using Senegalese sole (*Solea senegalensis*) fingerlings of approximately 15 g of weight. Fish were divided into four groups of 30 animals, one per tested strain, and were maintained into 50-L seawater tanks at 18°C with aeration and water recirculation. Colonies from a fresh 24-h plate were resuspended in saline solution (0.85% NaCl) to achieve an OD_600_ of 0.5. Fish were intraperitoneally injected with 100 μl of the bacterial suspension (2–3 × 10^3^ CFU/fish). The number of bacterial cells injected was determined by plating serial dilutions on TSA-1. A control group was injected with saline solution. Mortalities were followed for 12 days after injection and dead events were daily registered. Statistical differences in survival curves were determined using the Kaplan–Meier method with Mantel-Cox log-rank test using SPSS (version 20; IBM SPSS Inc., Chicago, IL). *p*-values were significant when *P* was < 0.05. The protocols for animal experimentation follow the current legislation and have been approved by the Bioethics Committee of the University of Santiago de Compostela.

### Promoter Sequences Analysis and Phylogenetic Reconstruction

To analyze the diversity of piscibactin gene promoters, we performed BlastN searches in the nucleotide collection (nr/nt) and whole-genome shotgun (wgs) NCBI databases using as a query the nucleotide sequence of *V. anguillarum* RV22 *irp*-HPI between 250 bp upstream *pbtA* and the *frpA* stop codon (sequence ID. AEZB01000030, from position 30,437 to 35,095). Homologous sequences were clustered in promoter types by similarity; thus, each type included sequences sharing 100% of coverage and≥99.5% of nucleotide identity. Representative sequences of each type were downloaded from NCBI and aligned using MUSCLE (MEGA X suite). Phylogenetic trees were inferred using p-distances (Transitions + Transversions) and Neighbor-Joining method. All ambiguous positions were removed for each sequence pair (pairwise deletion option). There was a total of 3,377 positions in the final dataset. Sequence alignments, nucleotide pairwise p-distances, and evolutionary analyses were conducted in MEGA X (Kumar et al., 2018).

## Results

### Deletion of *irp*-HPI Encoded AraC-Like Regulator PbtA Disables the Piscibactin System

To analyze the role of the two AraC-like regulators encoded by *irp*-HPI in piscibactin production, in-frame deletion mutants for *araC1* and *araC2* genes (Figure 1) were constructed in a *V. anguillarum* RV22Δ*vabF* background, a strain that produces only piscibactin as siderophore (Balado et al., 2018). Then, the RV22Δ*vabF* parental strain and its derivative Δ*araC1* and Δ*araC2* mutants were challenged to grow under iron excess (CM9 supplemented with FeCl_3_ 10 μM) and under iron-deprivation conditions by adding the iron chelator 2,2′-dipyridyl at 25 μM (weak iron-deprivation) or at 75 μM (strong iron-deprivation).

**FIGURE 2:**
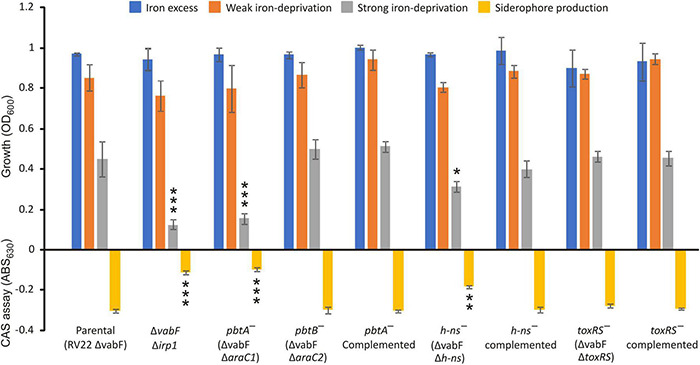
Growth ability under different iron availability conditions of *V. anguillarum* parental strain compared to its derivative *araC1*, *araC2*, *h-ns*, and *toxR-S* defective mutants and complemented strains. *V. anguillarum* strains were cultured in CM9 minimal medium supplemented with 10 μM FeCl_3_ (iron excess conditions), or the iron chelator 2,2′-dipyridyl at 25 μM (weak iron-deprivation), or at 75 μM 2,2′-dipyridyl (strong iron deprivation). Siderophore production was evaluated in cell-free supernatants after growth of each *V. anguillarum* strain in weak iron deprivation (CM9 with 25 μM 2,2′-dipyridyl) up to OD_600_ ca. 0.8. *t*-test was used to detect significant differences between each mutant and the parental strain. *p < 0.05; **p < 0.01; ***p < 0.001.

Under iron excess, or weak iron-deprivation conditions, no significant differences were observed between parental and mutant strains. However, under strong iron-deprivation conditions, the *ΔaraC1* defective mutant was impaired in growth (Figure 2). The *ΔaraC1* mutant showed a phenotype like that observed for the Δ*irp1* defective mutant that is unable to produce piscibactin (Balado et al., 2018). Under this condition, the parental strain and the Δ*araC2* defective mutant showed indistinguishable growth ability. Subsequent evaluation of siderophore content in the cell-free culture supernatants showed a strong reduction in the *ΔaraC1* defective mutant (Figure 2). When the Δ*araC1* mutant was complemented with a functional version of *araC1*, the parental phenotype was restored. This result suggests that *araC1* could encode a transcriptional regulator essential for siderophore piscibactin synthesis. Consequently, *araC1* and *araC2* were renamed as piscibactin transcriptional regulator *pbtA* (piscibactin regulator A) and *pbtB*, respectively.

**FIGURE 3:**
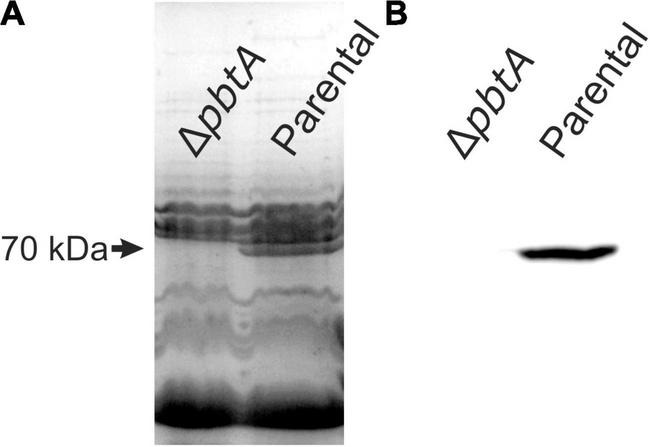
Outer membrane protein (OMP) band patterns **(A)** and detection of TonB-dependent piscibactin transporter FrpA by Western blot **(B)** in the *V. anguillarum* parental strain RV22Δ*vabF* and in the Δ*pbtA* (Δ*araC1*) mutant cultured under low-iron availability.

The piscibactin TonB-dependent outer membrane transporter *frpA* gene is located in the piscibactin operon upstream the genes encoding the biosynthetic functions (Balado et al., 2018; Figure 1). To determine whether inactivation of *pbtA* blocks the piscibactin siderophore system, the presence of FrpA in the *V. anguillarum ΔvabF* mutant (used as parental strain) and in its derivative *pbtA* defective mutant was determined by Western blot (Figure 3). While a unique protein band of ca. 70 kDa was detected (which is congruent with the 68-kDa molecular weight of FrpA) in the outer membrane sample (Figure 3A) of the *V. anguillarum* parental strain (RV22Δ*vabF*) and in the corresponding Western blot using anti-FrpA (Figure 3B), only a residual amount of FrpA was detected in the sample of the outer membrane proteins and Western blot of *V. anguillarum pbtA* defective mutant (Figure 3B). These findings greatly suggest that inactivation of the AraC-like transcriptional regulator PbtA disables iron uptake *via* the siderophore piscibactin.

### Inactivation of Both Global Regulators H-NS and ToxR-S Has a Slight Effect on *V. anguillarum* Growth Ability Under Low-Iron Conditions

To evaluate a putative role of the global regulators H-NS and ToxR-S in piscibactin production, single *h-ns* or *toxR-S* mutants were constructed. Then, growth ability and siderophore production were assayed in each mutant and compared to the parental strain RV22Δ*vabF* (Figure 2). While the phenotype of the Δ*toxR-S* mutant (RV22 Δ*vabF*Δ*toxR-S*) was indistinguishable from that of the parental strain, the Δ*h-ns* mutant showed a slight reduction of growth ability under strong iron-restricted conditions. The diminution of growth observed in the Δ*h-ns* mutant correlates with a decrease in piscibactin production. Finally, when the Δ*h-ns* mutant was complemented with a functional *h-ns* gene, the parental phenotype was restored. These results suggest that H-NS is required for a maximum piscibactin production. By contrast, ToxR-S did not exhibit a role in siderophore production.

### Inactivation of Either *pbtA* or *h-ns* Greatly Reduces *V. anguillarum* Virulence

**FIGURE 4:**
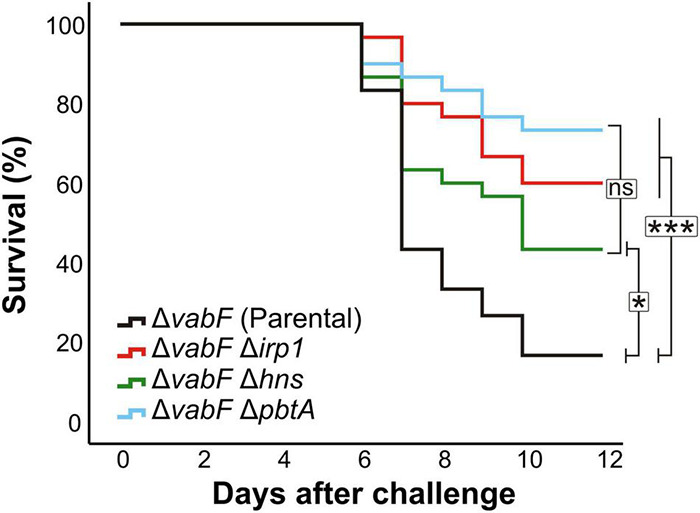
Survival abilities (Kaplan–Meier survival functions) of fish infected with the *V. anguillarum pbtA*^–^ (RV22 Δ*vabF*Δ*pbtA*) or *h-ns^–^* (RV22Δ*vabF*Δ*h-ns*) strains compared to parental strain (RV22Δ*vabF*) and the non-siderophore producer strain RV22Δ*vabF*Δ*irp1*. Asterisks denote statistical significance, * *p* < 0.05; *** *p* < 0.001, ns, no statistically significant differences.

To evaluate the role of PbtA and H-NS in *V. anguillarum* virulence, groups of 30 sole fingerlings were inoculated with a dose of 2–3 × 10^3^ CFU per fish of either *V. anguillarum* parental strain or one of its derivatives Δ*pbtA* or Δ*h-ns* mutant strains. A control group that was inoculated with saline solution did not show any signs of infection and no mortality was observed. The survival curves of each group of fish are shown in Figure 4. While the fish group challenged with the parental strain (RV22Δ*vabF*) showed a 60% mortality 7 days after infection, the *V. anguillarum pbtA* defective strain (Δ*pbtA*) showed a significant reduction of virulence since it caused approximately 20% mortality. Notably, survival curves of *pbtA* defective mutant and the *ΔvabFΔirp1* double mutant, impaired to produce siderophores (neither piscibactin nor vanchrobactin) (Balado et al., 2018), were statistically indistinguishable. In addition, inactivation of *h-ns* also resulted in a significant reduction of virulence for fish.

### The Expression of Piscibactin Biosynthetic and Transport Genes Is Under Control of *frpA* Promoter (P*frpA*) Whose Activity Depends on the AraC-Like Transcriptional Activator PbtA

**FIGURE 5:**
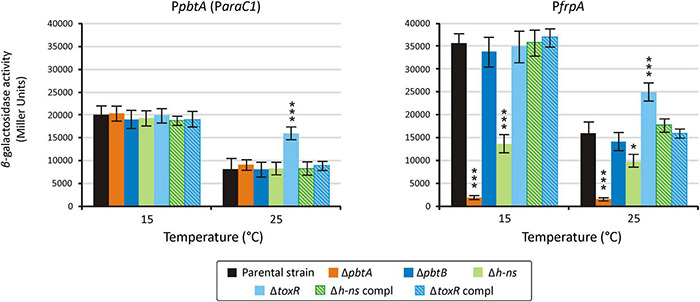
Transcriptional activity of the promoters P*pbtA* and P*frpA* in either *pbtA*, *pbtB, toxR-S*, and *h-ns* mutant strains measured under weak iron deprivation (CM9 supplemented with 25 μM 2,2′-dipyridyl). A *t*-test was used to detect significant differences between each mutant and the parental strain. * *p* < 0.05; *** *p* < 0.001.

Sequences upstream of *pbtA* (formerly *araC1*) and *frpA* (Figure 1) were previously characterized as the main promoter regions that control the expression of piscibactin synthesis and transport genes (Balado et al., 2018). Thus, to evaluate the role of the putative transcriptional regulator PbtA in the expression of piscibactin genes, the transcriptional activity of *pbtA* and *frpA* promoters (P*pbtA* and P*frpA*, respectively) was measured in a *pbtA*^–^ background (Δ*pbtA* mutant). At both temperatures tested, 15°C or 25°C, inactivation of *pbtA* results in the loss of P*frpA* activity (Figure 5). Notably, the expression pattern of P*pbtA* still follows a temperature-dependent pattern in a *pbtA*^–^ background, showing identical activity levels as the parental strain at the same temperatures. On the other hand, *pbtB* (formerly *araC2*) deletion did not alter the transcriptional levels of P*pbtA* or P*frpA.* P*pbtA* and P*frpA* promoter activity evaluation in the *pbtA*^–^ complemented strain could not be done since the plasmid used to complement (pSEVA651) and the plasmid used to obtain the LacZ fusions (pHRP309) both confer gentamicin resistance. However, as was shown above, complementation of Δ*pbtA* mutant with the wild-type gene restored a phenotype identical to the parental strain.

Transcriptional activity of P*pbtA* and P*frpA* was also evaluated in the *h-ns* and *toxR-S* defective mutants. Unexpectedly, while the activity of the *pbtA* promoter in the *h-ns* mutant was indistinguishable from that of the parental strain at both temperatures tested, the activity of *frpA* promoter (P*frpA*) in a Δ*h-ns* background was reduced ca. 60% at 15°C and ca. 20% at 25°C (Figure 4). The evaluation of piscibactin promoters activity in the Δ*toxR-S* mutant showed that the expression levels of P*pbtA* and P*frpA* are increased two-fold and 30%, respectively, at 25°C, when compared to the parental strain (Figure 5). No significant changes in expression were observed at 15°C. Complemented strains, either of *h-ns* or *toxR-S*, showed expression patterns indistinguishable from those of the parental strain.

### Different Versions of P*frpA* and P*pbtA* Promoters Are Present Within *Vibrionaceae* Members

**FIGURE 6:**
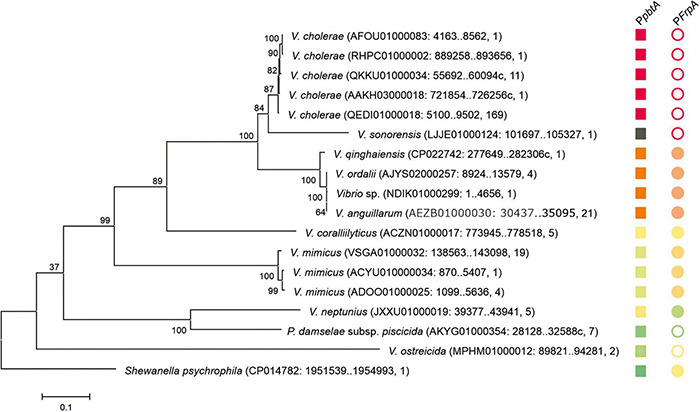
Phylogenetic relationships of the *pbtA-frpA* region of *irp*-HPI genomic island and distribution of P*pbtA* and P*frpA* piscibactin promoter versions. Sequences are identified with species name. Sequence ID, sequence region, and the number of closely related sequences deposited in GenBank sharing coverage of 100% and identity nucleotide sequence≥99.5% are shown between parentheses. The tree is drawn to scale, with branch lengths representing the evolutionary distances (number of base substitutions per site). The different versions of the piscibactin promoters P*pbtA* and P*frpA* are represented with squares and circles, respectively. Filled circles denote “long versions” of *frpA* promoter while empty circles denote “short versions.” Closely related promoter sequences, according to pairwise nucleotide p-distances (Supplementary Figures 3, 4) are represented with the same color. P*frpA* promoter of *V. sonorensis* is represented with a black square since it does not align with the other sequences.

**FIGURE 7:**
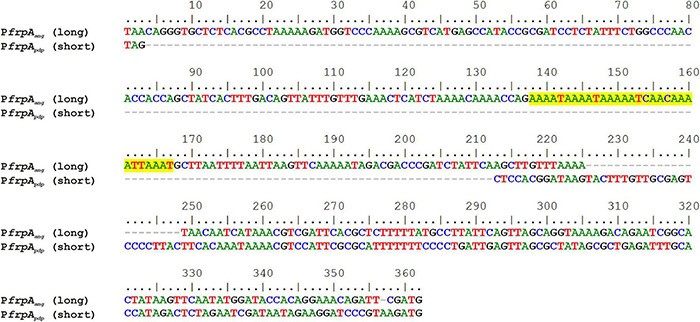
Alignment of a long and a short *pbtB*-*frpA* intergenic region (between *pbtB* stop codon and *frpA* ATG start codon) containing *frpA* promoter of *V. anguillarum* (P*frpA*_*ang*_) and *P. damselae* subsp. *piscicida* (P*frpA*_*pdp*_). Low complexity AAAAT repeat motifs are highlighted in yellow.

Several versions of the *irp*-HPI genomic island are found within *Vibrionaceae* showing identical gene structure and an overall nucleotide diversity (p-distance) of 0.3 substitutions per site (Figure 6). Not surprisingly, nucleotide diversity is much higher in intergenic regions than within protein-coding sequences. Alignment of representative *irp*-HPI genomic islands from different species showed that the sequences immediately upstream of *pbtA* ATG start codon (P*pbtA* region) (Supplementary Figure 1) and the *pbtB-frpA* intergenic region (P*frpA* region) (Supplementary Figure 2) showed major differences between *Vibrio* species and thus different types of piscibactin promoters P*pbtA* and P*frpA* could be defined according to their similarity. Notably, the distribution of each piscibactin promoter type (P*pbtA* and P*frpA* sequences) does not match with *irp*-HPI phylogenetic lineages (Figure 7). The most variable sequences are found in the *pbtB-frpA* intergenic region (Supplementary Figures 3,4). Although all *pbtB*-*frpA* sequences share a conserved region of ca. 100 bp located immediately upstream of *frpA* start codon, the region downstream of *pbtB* stop codon shows higher differences since deletions and/or insertions events would have occurred (Supplementary Figure 2). Thus, the *irp*-HPI genomic islands of species like *V. anguillarum*, *V. ordalli*, and *V. qinghaiensis* contain a long *pbtB*-*frpA* intergenic region with a size of ca. 360 bp, which includes a low complexity sequence between positions 137 and 167 with six repeats of an AAAAT motif (Figure 7 and Supplementary Figure 2). By contrast, *P. damselae*, *V. ostreicida, V. sonorensis*, and *V. cholerae* harbor shorter intergenic sequences with nucleotide lengths between 100 and 140 bp due to the lack of the segment immediately downstream of *pbtB* stop codon (Figure 7 and Supplementary Figure 1). In addition, there are some intermediate versions such as those found in *V. mimicus* and *V. neptunius* (Supplementary Figure 2). The high variability found upstream of *pbtA* (P*pbtA*) and in the *pbtB-frpA* intergenic region (P*frpA*) sequences could imply the existence of different expression patterns among those *Vibrio* spp. harboring the *irp*-HPI genomic island.

### *irp*-HPI Expression Pattern Results From the Interaction Between Recipient Genome Content and *pbtA* or *frpA* Promoter Type

**FIGURE 8:**
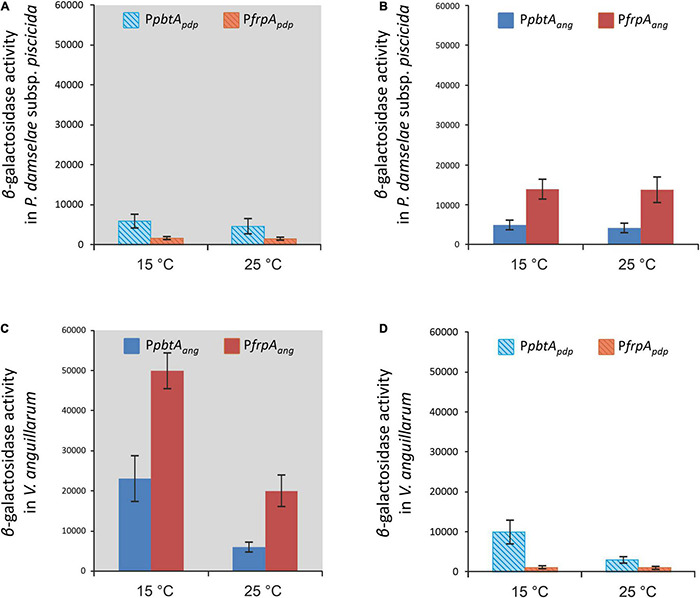
Native (**A,C**, gray background) and heterologous **(B,D)** transcriptional activity evaluation (β-galactosidase activity) of both versions of the piscibactin promoters P*frpA* (P*frpA*_*pdp*_ or P*frpA*_*ang*_) and P*pbtA* (P*pbtA*_*pdp*_ or P*pbtA*_*ang*_) in *P. damselae* subsp. *piscicida* **(A,B)** or in *V. anguillarum* **(C,D)** under iron restriction at 15 or 25°C.

To compare the expression pattern of different versions of the *irp*-HPI genomic island found in different *Vibrionaceae*, the expression pattern of the *irp*-HPI present in *Photobacterium damselae* subsp. *piscicida* (*irp*-HPI*_*pdp*_*) was compared with the expression pattern of the *V. anguillarum* island (*irp*-HPI*_*ang*_*). Particularly, *irp*-HPI*_*pdp*_* harbors the short version of *frpA* promoter (P*frpA*_*pdp*_) described above (Figures 6, 7). LacZ fusions of the sequences immediately upstream of *pbtA* and *frpA* from *P. damselae* subsp. *piscicida* (denoted as *pbtA*_*pdp*_ and *frpA*_*pdp*_, respectively), homologous to their counterparts of *V. anguillarum* (Figures 1, 7), were obtained and the transcriptional activity of each promoter (P*pbtA*_*pdp*_ and P*frpA*_*pdp*_) was assayed at 15 and 25°C under low iron availability (Figure 8A). The expression pattern of each piscibactin promoter from *P. damselae* subsp. *piscicida* (Figure 8A) showed extensive differences with piscibactin promoters from *V. anguillarum* (Figure 8C). Thus, P*pbtA*_*pdp*_ promoter is expressed in *P. damselae* subsp. *piscicida* although it showed almost the same transcriptional activity at 15°C and at 25°C (Figure 8A), which suggests that piscibactin genes, in contrast with the behavior described in *V. anguillarum* (Balado et al., 2018), do not show a temperature-dependent expression in *P. damselae* subsp. *piscicida*. In addition, P*pbtA*_*pdp*_ showed a threefold lower activity in *P. damselae* subsp. *piscicida* (Figure 8A) than its counterpart P*pbtA*_*ang*_ in *V. anguillarum* (Figure 8C). Unexpectedly, while P*frpA*_*ang*_ reached ca. 50,000 β-galactosidase units at 15°C, the activity displayed by P*frpA*_*pdp*_ was almost undetectable (<750 U), suggesting that the short *pbtA*-*frpA* intergenic region found in *irp*-HPI*_*pdp*_* does not contain a transcriptional promoter.

To ascertain whether the differences observed between the expression patterns of each version of *irp*-HPI promoters would be due to the differences in the promoter versions or to other factors encoded in the respective recipient genomes, a heterologous evaluation of each LacZ fusion was done (Figures 8B,D). The results showed that both versions of the *irp*-HPI promoters, either P*pbtA*_*pdp*_ and P*frpA*_*pdp*_ or P*pbtA*_*ang*_ and P*frpA*_*ang*_, have a 2.5-fold less transcriptional activity in *P. damselae* subsp. *piscicida* (Figures 8A,B) than P*pbtA*_*ang*_ and P*frpA*_*ang*_ in *V. anguillarum* (Figure 8C). The expression level achieved by P*pbtA*_*ang*_ in *P. damselae* subsp. *piscicida* was equivalent to that shown by the native evaluation of P*pbtA*_*pdp*_. Notably, while the transcriptional activity of P*pbtA*_*ang*_ and P*frpA*_*ang*_ measured within *P. damselae* subsp. *piscicida* did not show differences between temperatures (Figure 8B), the P*pbtA*_*pdp*_ promoter showed a three-fold higher transcriptional activity at 15°C than at 25°C when its activities were measured within *V. anguillarum* (Figure 8D). By contrast, P*frpA_*p*__*d*__*p*_* did not show any activity within *V. anguillarum*, which greatly suggests that the short version of *pbtB-frpA* intergenic sequence present in *irp*-HPI*_*pdp*_* does not contain a transcriptional promoter (Figure 8A).

## Discussion

Horizontally acquired DNA usually encodes transcriptional regulators that promote their own expression (Stoebel et al., 2008). To ascertain the regulatory mechanism that controls the expression of *irp*-HPI genes, we constructed and analyzed *V. anguillarum* mutants defective in the two *irp*-HPI encoded AraC-like regulators PbtA and PbtB, and in the global regulators H-NS and ToxR-S. Inactivation of *pbtA* (*araC1*) disables piscibactin biosynthesis and transport, greatly reducing growing capacities under low iron availability. It also resulted in a marked decrease on the degree of virulence, confirming the relevance of piscibactin synthesis in the pathogenesis of *V. anguillarum* (Balado et al., 2018). By contrast, neither phenotypic nor piscibactin gene expression changes were found by the inactivation of *pbtB* (*araC2*). Generally, the regulation of iron uptake systems in Gram-negative bacteria is mediated by the negative transcriptional regulator Fur (ferric uptake regulator) (Fillat, 2014). Conversely, some AraC-like transcriptional regulators have been identified as exerting positive regulation in the expression of the siderophore biosynthetic and transport genes such as AlcR in *Bordetella pertussis* that upregulates the expression of alcaligin siderophore biosynthesis and transport (Brickman et al., 2001), the ferrioxamine B transport regulator DesR in *V. vulnificus* (Tanabe et al., 2005), or PchR in *Pseudomonas aeruginosa*, which is required for maximal expression of the pyochelin biosynthetic and transport genes (Michel et al., 2005).

AraC-like regulators can act as autoinducers by inducing its own expression, and/or activating the expression of other promoters *in trans* (Gallegos et al., 1997; Galperin, 2006). The piscibactin system is transcribed within a large operon whose transcription could start from alternative promoters located upstream of *pbtA* (*araC1*) and *frpA* (Figure 1; Balado et al., 2018). The piscibactin siderophore system is structurally and functionally related to the yersiniabactin system (Osorio et al., 2006; Souto et al., 2012). In *Yersinia pestis* and *E. coli*, the AraC-like protein named YbtA, encoded in the yersiniabactin gene cluster, activates the expression of yersiniabactin genes, including its own promoter (Fetherston et al., 1996; Anisimov et al., 2005); however, the AraC-like PbtA described here does not enhance the expression of its own promoter (P*pbtA*). Although deletion of *ybtA* blocks yersiniabactin production, the *ybtA* mutant has a virulence phenotype intermediate between the wild type and a yersiniabactin biosynthesis null mutant (Smati et al., 2017). Our results clearly show that inactivation of PbtA in *V. anguillarum* results in an attenuation of the degree of virulence equivalent to that observed in a *V. anguillarum* strain unable to produce siderophores (RV22Δ*vabF*Δ*irp1*). This finding, together with the absence of FrpA in the outer membrane of the *V. anguillarum pbtA*^–^ strain and the extremely low levels of transcriptional activity found in P*frpA* promoter, strongly suggests that (1) PbtA is a transcriptional activator required to express the transport and biosynthesis components of the piscibactin siderophore system, (2) effective expression of piscibactin functions only occurs from *frpA* promoter (P*frpA*), and (3) the piscibactin regulator PbtA plays a key role in *V. anguillarum* virulence.

The AraC-like regulators can be part of a larger regulon that can act in a cascade manner as a response to environmental signals. ToxR is a regulatory protein that is essential for virulence in a range of different pathogenic vibrios (Herrington et al., 1988; Peterson and Mekalanos, 1988; Lee et al., 2000; Whitaker et al., 2012). In *Vibrio cholerae*, the ToxR regulon is responsible for the transcriptional activation of the AraC-like regulator ToxT, which responds to environmental signals to directly activate many virulence-related genes (Higgins and DiRita, 1994; Skorupski and Taylor, 1997; Childers and Klose, 2007). However, some works suggest that in *V. anguillarum*, ToxR is not a major regulator of virulence factors (Okuda et al., 2001; Wang et al., 2002). Our results showed that deletion of the global regulator ToxR-S system in *V. anguillarum* did not cause appreciable changes in growth ability under iron restriction nor apparently had effects on siderophore production at cold temperatures. Although ToxR-S could have an indirect effect in piscibactin genes expression at warm temperatures, *irp*-HPI would not be part of the ToxR regulon.

Gram-negative bacteria possess tools to silence the expression of horizontally acquired genes such as the global repressor H-NS, which is implicated in xenogenetic silencing (Stoebel et al., 2008; Prajapat and Saini, 2012). The *V. anguillarum* H-NS mutant strain showed a slight reduction in growth potential under low-iron conditions, which is the result of a lower activity of the piscibactin promoter P*frpA* at both cold and warm temperatures (15 and 25°C). Thus, our results suggest that H-NS must exert an indirect effect on the expression of piscibactin biosynthetic and transport genes. However, the transcriptional activity of P*pbtA* promoter does not change when H-NS is inactivated. Therefore, H-NS would not have any direct or indirect regulatory effect on the expression of the piscibactin transcriptional activator PbtA. Although H-NS can also mediate processes of temperature-dependent expression regulation by upregulating target genes when temperature increases (Mou et al., 2013), this type of regulatory effect was not observed in our data. Thus, although H-NS would not mediate in the temperature-dependent modulation of piscibactin genes, it is required to achieve the maximum activity of piscibactin promoter P*frpA* (Figure 5). Consequently, and since our previous works demonstrated that piscibactin has a great impact in *V. anguillarum* virulence (Balado et al., 2018), inactivation of *h-ns* results in a significant reduction of *V. anguillarum* virulence for fish (Figure 4).

The role of evolutionary dynamics in bacterial disease is not well understood, but it is known that intensive aquaculture selects for increased virulence, which may trigger the emergence of novel diseases (Pulkkinen et al., 2010; Sundberg et al., 2016). In addition, the rapid spread of selectively favored virulence factors by horizontal gene transfer (HGT) facilitates the emergence of new bacterial diseases (Bruto et al., 2017; Le Roux and Blokesch, 2018). However, horizontally transferred genes are not always expressed in the recipient genome, because of possible incompatibilities in promoter sequences, different codon usages, and/or excessive energy cost (Ochman et al., 2000; Park and Zhang, 2012). They must be subjected to the precise regulatory control by the recipient’s genome that allows genes to be expressed under the control of specific signals (Stoebel et al., 2008). The piscibactin high pathogenicity island (*irp*-HPI) is widespread among *Vibrionaceae* including the human pathogen *V. cholerae* (Thode et al., 2018). Phylogenetic analysis clearly shows that several *irp*-HPI lineages exist and that extensive sequence differences are present in the intergenic regions where piscibactin promoters P*pbtA* and P*frpA* are found. The piscibactin siderophore system was firstly identified in the marine pathogen *P. damselae* subsp. *piscicida* and it is also a major virulence factor in this bacterium (Osorio et al., 2006, 2015). However, the expression pattern of *irp*-HPI genes described in *V. anguillarum* is almost incompatible with the ecology of *P. damselae* subsp. *piscicida*, which mainly cause disease outbreaks when water temperature is above 20°C (Romalde, 2002; Toranzo et al., 2005; Bellos et al., 2015). Notably, *P. damselae* subsp. *piscicida irp*-HPI genomic island (*irp*-HPI*_*pdp*_*) lacks a promoter in *frpA*. Thereby, all piscibactin genes must be necessarily expressed from the *pbtA* promoter (P*pbtA*) in a large operon that includes a regulator, and biosynthetic and transport functions (Figure 1; Osorio et al., 2006). Interestingly, *irp*-HPI*_*pdp*_* did not follow a temperature-dependent expression pattern in *P. damselae* subsp. *piscicida* and the transcriptional activity is significantly lower than in *V. anguillarum*, but its expression is activated at low temperatures when it is inserted in *V. anguillarum*. Conversely, the transcriptional activity of *V. anguillarum* promoters P*pbtA* and P*frpA* do not respond to temperature changes when they are inserted in *P. damselae* subsp. *piscicida*. Thus, *irp*-HPI temperature-dependent expression pattern would be modulated by yet unknown activator(s) present in the recipient genome, which would enhance transcription of P*pbtA* when temperature decreases, rather than by an inherent property of the *irp*-HPI genomic island.

As a conclusion, the temperature-dependent expression pattern of the piscibactin-encoding high-pathogenicity island *irp*-HPI described in *V. anguillarum* is the result of the combined effect of the AraC-like transcriptional activator PbtA encoded by this genomic island and regulatory factor(s) encoded by the recipient bacterial genome. Thus, the horizontally acquired piscibactin genes would have been subjected to global cell control, maximizing bacterial fitness advantages (Stoebel et al., 2008). Notably, the existence of different expression patterns within different *irp*-HPI evolutionary lineages supports the hypothesis of the long-term evolution of the *irp*-HPI genomic islands within the *Vibrionaceae* (Thode et al., 2018). The mechanism that modulates piscibactin gene expression through PbtA and/or the putative regulatory effect(s) exerted by PbtA outside the genomic island could result in global modulation of virulence factors in response to temperature changes. Thus, further studies focused on the PbtA regulation mechanism will be highly valuable to decipher the adaptation of *V. anguillarum* and other members of *Vibrionaceae* to environmental temperature changes.

## Data Availability Statement

The original contributions presented in the study are included in the article/Supplementary material, further inquiries can be directed to the corresponding author/s.

## Ethics Statement

The animal study was reviewed and approved by the Bioethics Committee of the University of Santiago de Compostela, Spain.

## Author Contributions

MAL and MB performed the lab experiments, analyzed the data, and wrote the first draft of the manuscript. MB and MLL corrected the draft and built the final version of the manuscript. All authors conceived and designed the study, contributed to manuscript revision, and read and approved the submitted version.

## Conflict of Interest

The authors declare that the research was conducted in the absence of any commercial or financial relationships that could be construed as a potential conflict of interest.

## Publisher’s Note

All claims expressed in this article are solely those of the authors and do not necessarily represent those of their affiliated organizations, or those of the publisher, the editors and the reviewers. Any product that may be evaluated in this article, or claim that may be made by its manufacturer, is not guaranteed or endorsed by the publisher.
