# Supplementary material for: The Temperature-Dependent Expression of the High-Pathogenicity Island Encoding Piscibactin in Vibrionaceae Results From the Combined Effect of the AraC-Like Transcriptional Activator PbtA and Regulatory Factors From the Recipient Genome
Source: Front Microbiol. 2021 Nov 19;12:748147. doi: 10.3389/fmicb.2021.748147 (PMC8639528; doi:10.3389/fmicb.2021.748147)
Supplement: Supplementary file 1 [file Data_Sheet_1.PDF]

SUPPLEMENTARY INFORMATION

The temperature-dependent expression of the high-pathogenicity island encoding piscibactin in *Vibrionaceae* results from the combined effect of the AraC-like transcriptional activator PbtA and regulatory factors from the recipient genome

Marta A. Lages, Manuel L. Lemos*, Miguel Balado*

Department of Microbiology and Parasitology, Institute of Aquaculture, Universidade de Santiago de Compostela, Santiago de Compostela 15782, Spain.

Table S1. Oligonucleotides used for construction of mutants by allele exchange, complementation of mutants and transcriptional *lacZ* fusions.

Figure S1. Alignment of representative versions of P*pbtA* promoter regions (250 bp upstream *pbtA* ATG start).

Figure S2. Alignment of representative versions of *pbtB*(*araC2)-frpA* intergenic region (between *pbtB* stop codon and *frpA* ATG start codon).

Figure S3. Pairwise nucleotide p-distances (Transitions + Transversions) of P*pbtA* promoter regions.

Figure S4. Pairwise nucleotide p-distances (Transitions + Transversions) of P*frpA* promoter regions.

Table S1. Oligonucleotides used for construction of mutants by allele exchange, complementation of mutants and transcriptional *lacZ* fusions. Recognition sequences for restriction enzymes are underlined.

Oligonucleotide (5' -> 3')		Amplification size (bp)
araC1 mutant construction		
AraC1_1_EcoRI	GCGGAATTCTACAGCAAAGAGTGGAACGG	927
AraC1_2_BamHI	GCGGGATCCCGGTAAGCGTATGGATGTGG	
AraC1_3_BamHI	GCGGGATCCCGTCCGATTATCGACATCGC	945
AraC1_4_XbaI	GCGTCTAGATGTTGTTGAGTGCTGCCATC	
araC2 mutant construction		
AraC2_1_EcoRI	GCGGAATTCTGAGTGAGAGTTTGCGTGAC	903
AraC2_2_BamHI	CGCGGATCCGATCACACAGCAACGTAACG	
AraC2_3_BamHI	GGCGGATCCCTCAGCGCAGAAATCTACAC	954
AraC2_4_XbaI	CGCTCTAGATTGAGGTTACTGTAGCTCGC	
h-ns mutant construction		
1_HNS_F_XbaI	CGCTCTAGAGCGCCTAATACAATACCGA	888
2_HNS_R_BamHI	GCGGGATCCAGCGCGTAGGCTACGAATAT	
3_HNS_F_BamHI	CCGGGATCCCAAGAGCAACTTGATGCAGG	800
4_HNS_R_XhoI	GGCCTCGAGCCAATGCCAGAGGCAACAAT	
toxR-S mutant construction		
1_ToxR_F_XbaI	CCGTCTAGAAGCGCGAGTTCTTACAGTGA	746
2_ToxR_R_PstI	GCGCTGCAGCGTGAATCGGTTAGCAAGGA	
3_ToxS_F_PstI	GGCCTGCAGGTCGACGTATTGATGTCGTC	626
4_ToxS_R_XhoI	GCCCTCGAGGCGCAAGGTCAAGATGATGT	
araC1 complementation		
AraC1_comp_F_XbaI	CGGTCTAGACTGATCTCTGGAGATAGAGC	1537
AraC1_comp_R_BamHI	CCGGGATCCATGACTTCACCAGCACGACT	
h-ns complementation		
HNS_comp_F_NotI	CCGGCGGCCGCCGACAGAGGAGATGATGCAA	1329
HNS_comp_R_ApaI	GCGGGGCCCTCCAACCGTGCCAGTTATGA	
toxR-S complementation		
ToxR-S_comp_F_NotI	CGCGCGGCCGCCGTATTCGCTTGGGTATCAA	2101
ToxR-S_comp_R_ApaI	GCGGGGCCCAGGGTAGAGACGCACAGATT	
P*frpA*$_{pdp}$ promoter fusion construction		
frpA_pF_XbaI	CGCTCTAGACGATAGCTTAGCGACATCTC	958
frpA_pR_BamHI	GGCGGATCCAGGGCTCAAGGTTGCAGCTA	
P*araC1*$_{pdp}$ promoter fusion construction		
araC1_pF_XbaI	GGCTCTAGAACGTTAACTCGCCAACTGCT	1056
araC1_pR_BamHI	CGCGGATCCTGGTGTTGTTGCACCAGAGC	

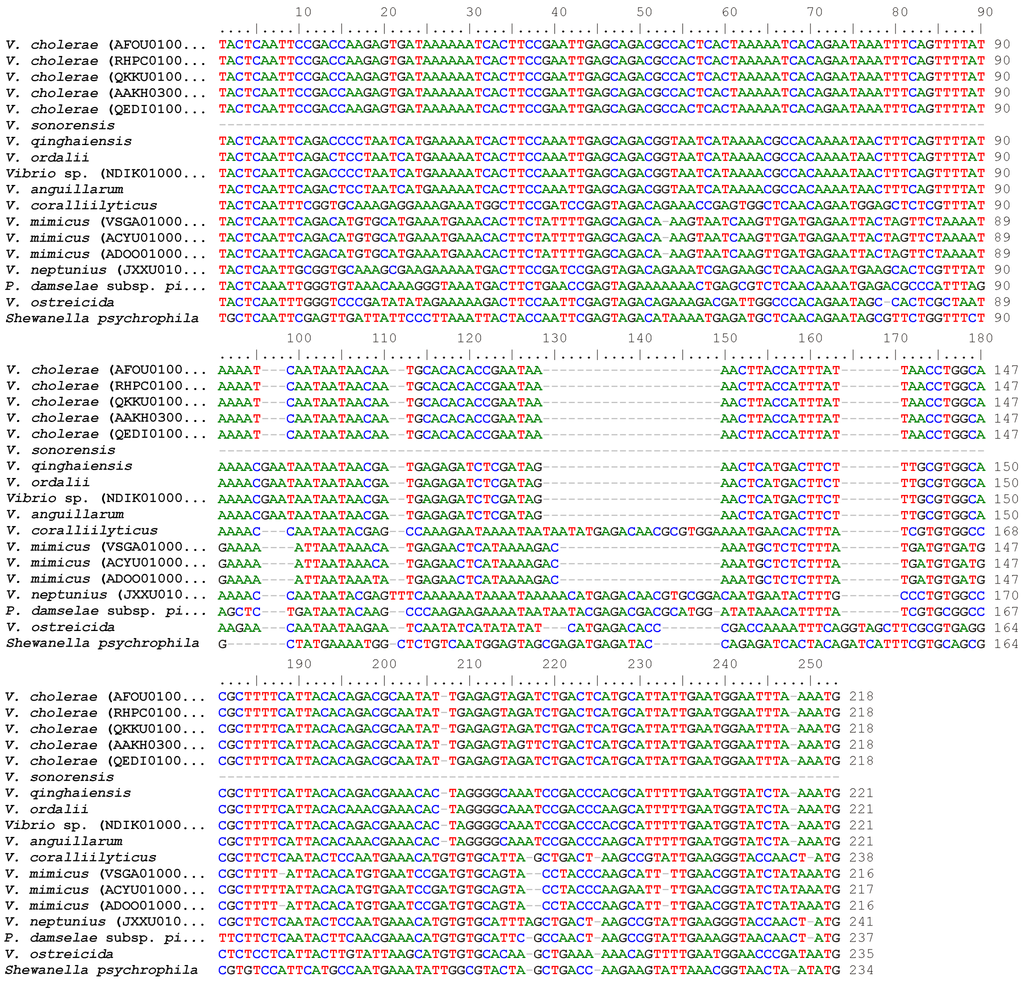

Figure S1. Alignment of representative versions of P*pbtA* promoter regions (250 bp upstream *pbtA* ATG start).

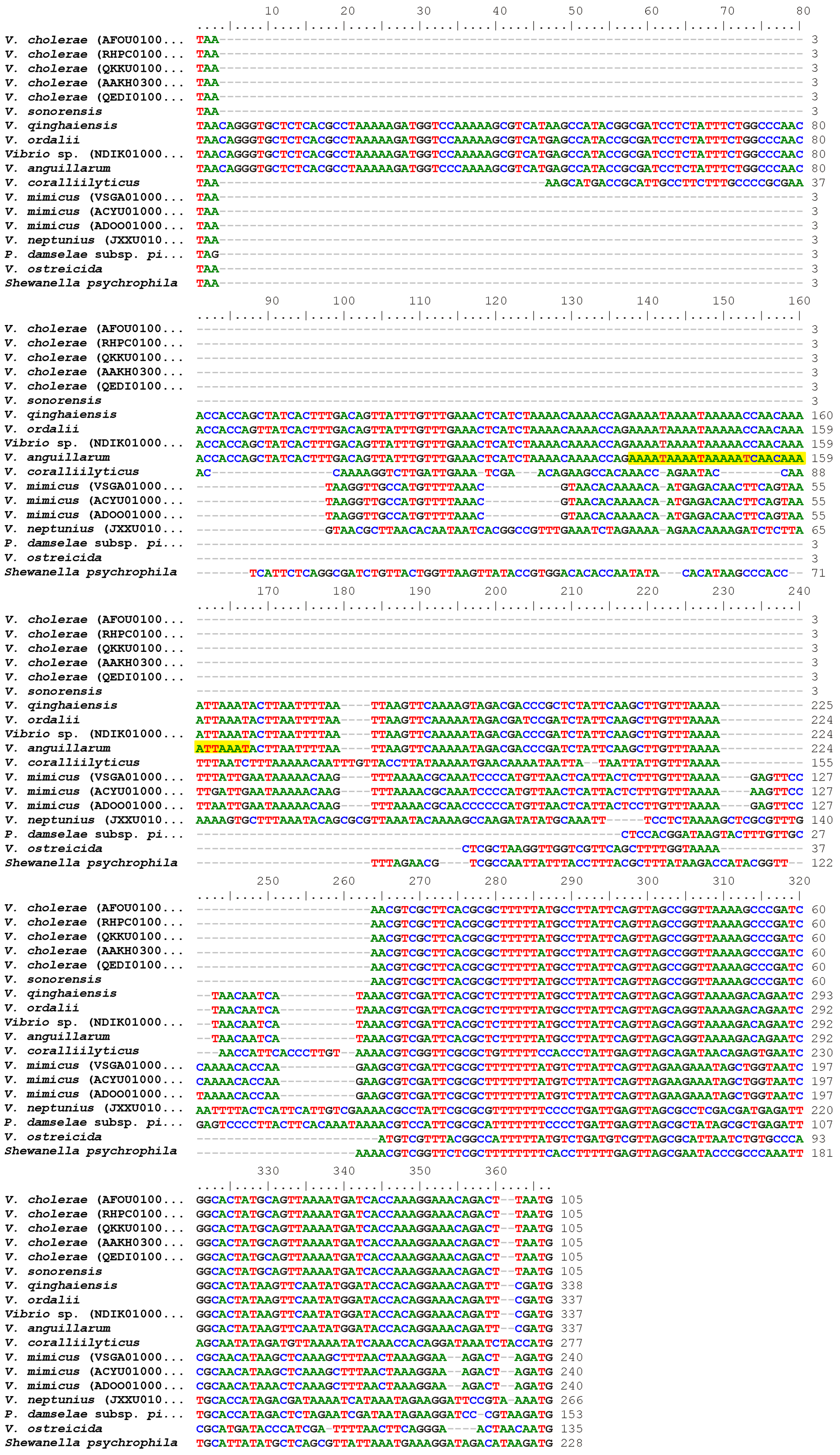

Figure S2. Alignment of representative versions of *pbtB*(*araC2)-frpA* intergenic region (between *pbtB* stop codon and *frpA* ATG start codon). Low complexity AAAAT repeat motifs are highlighted in yellow.

	V. cholerae (AFOU0100...	V. cholerae (RHPC0100...	V. cholerae (QKKU0100...	V. cholerae (AAKH0300...	V. cholerae (QEDI0100...	V. sonorensis (LJJE01...	V. qinghaiensis (CP02...	V. ordalii (AJYS02000...	Vibrio sp. (NDIK01000...	V. anguillarum	V. coralliilyticus	V. mimicus (VSGA01000...	V. mimicus (ACYU01000...	V. mimicus (ADOO01000...	V. neptunius	P. damselae subsp. pi...	V. ostreicida
V. cholerae **(RHPC0100...**	0.00																
V. cholerae **(QKKU0100...**	0.00	0.00															
V. cholerae **(AAKH0300...**	0.00	0.00	0.00														
V. cholerae **(QEDI0100...**	0.00	0.00	0.00	0.00													
V. sonorensis **(LJJE01...**																	
V. qinghaiensis **(CP02...**	0.24	0.24	0.24	0.24	0.25												
V. ordalii **(AJYS02000...**	0.25	0.25	0.25	0.25	0.26		0.01										
Vibrio **sp. (NDIK01000...**	0.24	0.24	0.24	0.24	0.25		0.00	0.01									
V. anguillarum	0.25	0.25	0.25	0.25	0.26		0.01	0.00	0.01								
V. coralliilyticus	0.40	0.40	0.40	0.40	0.40		0.41	0.40	0.41	0.40							
V. mimicus **(VSGA01000...**	0.42	0.42	0.42	0.42	0.42		0.36	0.36	0.36	0.36	0.43						
V. mimicus **(ACYU01000...**	0.42	0.42	0.42	0.42	0.42		0.36	0.36	0.36	0.36	0.43	0.00					
V. mimicus **(ADOO01000...**	0.43	0.43	0.43	0.43	0.43		0.37	0.37	0.37	0.37	0.44	0.01	0.00				
V. neptunius	0.38	0.38	0.38	0.38	0.38		0.39	0.38	0.39	0.38	0.09	0.43	0.43	0.44			
P. damselae **subsp. *pi*...**	0.48	0.48	0.48	0.48	0.48		0.47	0.46	0.47	0.46	0.21	0.51	0.51	0.52	0.25		
V. ostreicida	0.45	0.45	0.45	0.45	0.45		0.41	0.41	0.41	0.41	0.33	0.46	0.46	0.47	0.34	0.43	
Shewanella psychrophila	0.50	0.50	0.50	0.50	0.50		0.46	0.44	0.46	0.44	0.38	0.50	0.50	0.50	0.39	0.46	0.47

Figure S3. Pairwise nucleotide p-distances (Transitions + Transversions) of P*pbtA* promoter regions.

	V. cholerae (AFOU0100...	V. cholerae (RHPC0100...	V. cholerae (QKKU0100...	V. cholerae (AAKH0300...	V. cholerae (QEDI0100...	V. sonorensis (LJJE01...	V. qinghaiensis (CP02...	V. ordalii (AJYS02000...	Vibrio sp. (NDIK01000...	V. anguillarum	V. coralliilyticus	V. mimicus (VSGA01000...	V. mimicus (ACYU01000...	V. mimicus (ADOO01000...	V. neptunius	P. damselae subsp. pi...	V. ostreicida
***V. cholerae* (RHPC0100...**	0.00																
***V. cholerae* (QKKU0100...**	0.00	0.00															
***V. cholerae* (AAKH0300...**	0.00	0.00	0.00														
***V. cholerae* (QEDI0100...**	0.00	0.00	0.00	0.00													
***V. sonorensis* (LJJE01...**	0.00	0.00	0.00	0.00	0.00												
***V. qinghaiensis* (CP02...**	0.21	0.21	0.21	0.21	0.21	0.21											
***V. ordalii* (AJYS02000...**	0.21	0.21	0.21	0.21	0.21	0.21	0.01										
***Vibrio* sp. (NDIK01000...**	0.21	0.21	0.21	0.21	0.21	0.21	0.01	0.00									
V. anguillarum	0.21	0.21	0.21	0.21	0.21	0.21	0.01	0.00	0.00								
V. coralliilyticus	0.34	0.34	0.34	0.34	0.34	0.34	0.41	0.40	0.40	0.40							
***V. mimicus* (VSGA01000...**	0.40	0.40	0.40	0.40	0.40	0.40	0.41	0.40	0.40	0.40	0.42						
***V. mimicus* (ACYU01000...**	0.34	0.34	0.34	0.34	0.34	0.34	0.42	0.41	0.41	0.41	0.00	0.43					
***V. mimicus* (ADOO01000...**	0.33	0.33	0.33	0.33	0.33	0.33	0.42	0.42	0.42	0.42	0.02	0.43	0.02				
V. neptunius	0.47	0.47	0.47	0.47	0.47	0.47	0.49	0.49	0.49	0.49	0.50	0.47	0.50	0.49			
***P. damselae* subsp. *pi*...**	0.49	0.49	0.49	0.49	0.49	0.49	0.53	0.53	0.53	0.53	0.51	0.45	0.51	0.49	0.26		
V. ostreicida	0.42	0.42	0.42	0.42	0.42	0.42	0.49	0.49	0.49	0.49	0.41	0.50	0.41	0.41	0.54	0.56	
Shewanella psychrophila	0.43	0.43	0.43	0.43	0.43	0.43	0.46	0.46	0.46	0.46	0.46	0.46	0.46	0.47	0.45	0.37	0.50

Figure S4. Pairwise nucleotide p-distances (Transitions + Transversions) of P*frpA* promoter regions.
